# Structural Imaging-Based Biomarkers for Detecting Craving and Predicting Relapse in Subjects With Methamphetamine Dependence

**DOI:** 10.3389/fpsyt.2020.599099

**Published:** 2021-01-15

**Authors:** Chang Qi, Xiaobing Fan, Sean Foxley, Qiuxia Wu, Jinsong Tang, Wei Hao, An Xie, Jianbin Liu, Zhijuan Feng, Tieqiao Liu, Yanhui Liao

**Affiliations:** ^1^Department of Psychiatry, Zhejiang Provincial People's Hospital, People's Hospital of Hangzhou Medical College, Hangzhou, China; ^2^Department of Radiology, The University of Chicago, Chicago, IL, United States; ^3^Department of Psychiatry, The Second Xiangya Hospital, Central South University, Changsha, China; ^4^Department of Psychiatry, School of Medicine, Sir Run Run Shaw Hospital, Zhejiang University, Hangzhou, China; ^5^Key Laboratory of Medical Neurobiology of Zhejiang Province, Hangzhou, China; ^6^Department of Radiology, The People's Hospital of Hunan Province, Changsha, China; ^7^Department of Psychiatry, Weifang Mental Health Center, Weifang, China

**Keywords:** structural imaging, biomarkers, craving, relapse, methamphetamine use

## Abstract

**Background:** Craving is the predictor of relapse, and insula cortex (IC) is a critical neural substrate for craving and drug seeking. This study investigated whether IC abnormalities among MA users can detect craving state and predict relapse susceptibility.

**Methods:** A total of 142 subjects with a history of MA dependence completed structural MRI (sMRI) scans, and 30 subjects (10 subjects relapsed) completed 4-month follow-up scans. MA craving was measured by the Visual Analog Scale for Craving. Abnormalities of IC gray matter volume (GMV) between the subjects with and without craving were investigated by voxel-based morphometry (VBM). The receiver operating characteristic (ROC) analysis was performed for the region-of-interest (ROI) of IC GMV to assess the diagnostic accuracy.

**Results:** By comparing whole-brain volume maps, this study found that subjects without craving (*n* = 64) had a significantly extensive decrease in IC GMV (family-wise error correction, *p* < 0.05) than subjects with craving group (*n* = 78). The ROI of IC GMV had a significantly positive correlation with the craving scores reported by MA users. The ROC analysis showed a good discrimination (area under curve is 0.82/0.80 left/right) for IC GMV between the subjects with and without craving. By selecting Youden index cut-off point from whole model group, calculated sensitivity/specificity was equal to 78/70% and 70/75% for left and right IC, respectively. By applying the above optimal cut-off values to 30 follow-up subjects as validations, the results showed a similar sensitivity (73–80%) and specificity (73–80%) for detecting craving state as model group. For predicting relapse susceptibility, the sensitivity (50–55%) was low and the specificity (80–90%) was high.

**Conclusions:** Our study provides the first evidence that sMRI may be used to diagnosis the craving state in MA users based on optimal cut-off values, which could be served as MRI bio-markers and an objective measure of craving state.

## Introduction

Drug addiction is a chronic brain disease marked by high rates of relapse even after months or years of abstinence, long after self-reported craving and withdrawal have abated (1–3). The psychostimulant methamphetamine (MA) is a highly addictive and widely abused drug that has no available pharmacotherapies at the present (4). A core challenge for treating MA and other drug addiction is the high propensity for relapse. Relapse prevention treatment should be aimed at helping to control craving (5). However, reliable and valid biomarkers that accurately predict relapse and developmental processes in craving are still not available. These facts highlight the need for further insight into the neurobiology of drug craving might be gained by exploiting variations in the magnitude of cravings, which will facilitate appropriated medical interventions to prevent relapse among drug dependent individuals.

Since the report of being able to stop smoking without cravings by insular lesions (due to stroke) among smoking patients, extensive evidence indicates that drug addiction and craving have been associated with alterations in the insula cortex (IC). The IC has a key role in rebalancing the homeostatic imbalance, such as deprivation from drugs and reward cues (6, 7). The essential role of the insula in craving across drug categories provides a strong mechanistic foundation for exploring structural imaging-based biomarkers for detecting craving in methamphetamine addiction (6–8). Furthermore, exploring the neural correlates of IC as a predictor of relapse and craving state will provide the basis for future treatment strategies of relapse prevention.

Neuroimaging markers play an important role in gaining a deeper insight into the neurobiological basis of drug addiction. Structural brain abnormally among long-term drug users may reflect pathophysiologic processes of the addiction which may in turn act as treatment response markers to guide treatment options. The role of the insula in addiction and craving is evident. However, it is still unclear whether morphological alterations of the insular cortex constitute a state characteristic of the addiction that is present only during the course of the addictive disorder, or a trait marker that underlies a neurobiological vulnerability to the addiction that is present even during periods of sobriety. Investigating the individuals currently present subjective craving for MA and those currently without subjective craving for MA may be a particularly useful approach to shed light on this issue or to provide a necessary step toward identifying addiction traits. Thus, we assessed whether IC could be used as a predictor of relapse and craving state in this study.

## Materials and Methods

### Subjects

A total of 142 subjects with a history of MA use from two rehabilitation centers (the Kangda Voluntary Drug Rehabilitation Center in Hunan province and the Department of Addiction Medicine, Hunan Brain Hospital) were recruited between June 2013 to June 2015. Inclusion criteria: (1) aged between 18 and 42 years old; (2) being able to give voluntary informed consent; (3) met the Diagnostic and Statistical Manual of Mental Disorders, Fourth Edition (DSM-IV) (9) criteria for MA dependence (MAD); and (4) had no history of neurological illness or other major physical diseases. Exclusion criteria: (1) met the DSM-IV criteria for any other substance dependence (excluding nicotine) at any time; (2) presence of a major medical or neurological illness; (3) a lifetime history of a psychotic disorder; (4) a history of traumatic head injury, pregnancy; or (5) contraindications for MRI.

The study protocol was approved by the Ethics committee of the Second Xiangya Hospital, Central South University, Hunan, China (No. S095, 2013) and carried out in accordance with the Declaration of Helsinki.

### Procedures

All subjects underwent clinical and drug use history assessments by two qualified psychiatrists. All subjects were diagnosed for MA dependence with the Structured Clinical Interview for DSM-IV (9). Visual Analog Scale for Craving (VASc) (10) was used to measure MA craving. Subjects scored their craving by VASc right before the MRI scan on a scale from 0 (no craving) to 10 (extreme craving). The “craving group” was defined as subjects who had craving scores ≥ 1, and the “without craving group” was defined as subjects who had craving scores = 0. To maintain sobriety during the program, subjects were screened for the presence of drugs via urine toxicology. Study procedures occurred after 2 weeks of detoxification treatment. All subjects (*n* = 142) were given written informed consent to participate in a clinical interview session, the first MRI session, and a follow-up phone interview at 4-month to invite subjects (*n* = 30) for the second MRI session. At follow-up visit, all subjects were asked about their current living and mental situation and whether they had used any of the following substances in the past months: hallucinogens, stimulants, ketamine, or heroin (confirmed by a urine test). Relapse was defined as any use of these substances. During the follow-up, 20 subjects remained abstinent from drugs (with the exception of nicotine) from the time of treatment to 4-month follow-up, 10 subjects relapsed (mainly used MA).

The timeline/flowchart the study design is shown in the following:


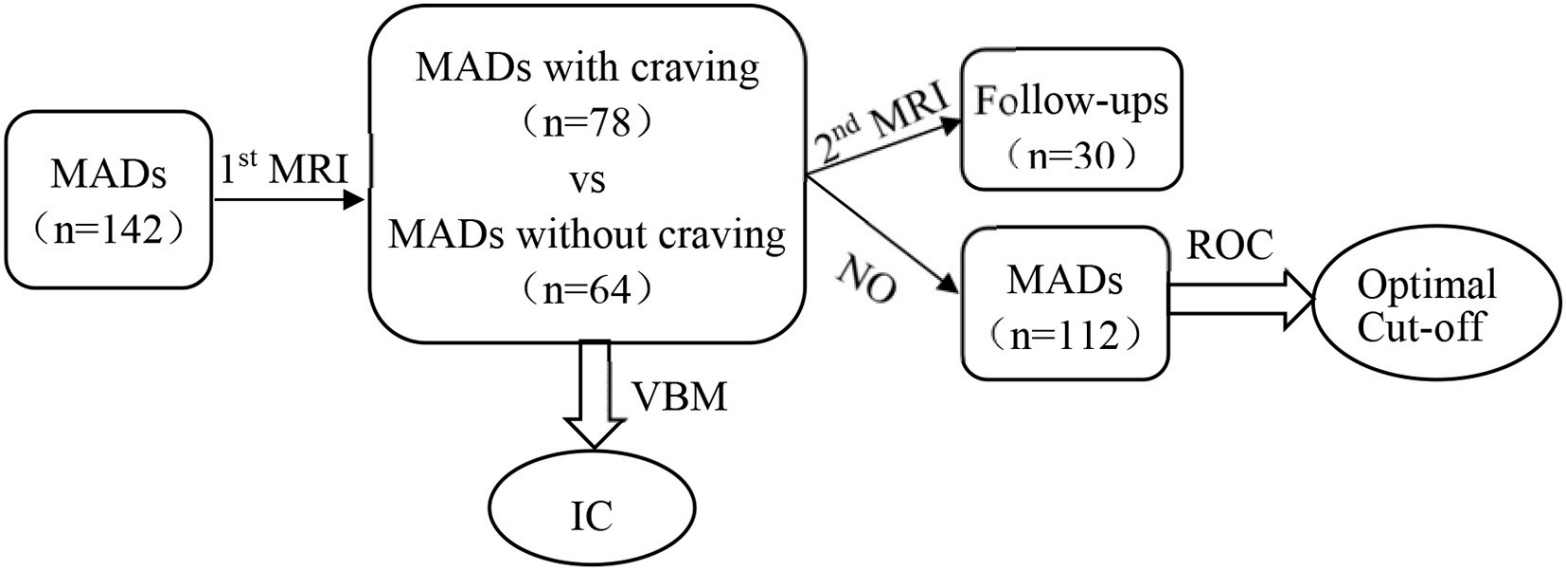


### MRI Data Acquisition

All MRI data were acquired with a 3.0-T Siemens Magentom Trio scanner (Siemens, Erlangen, Germany) at the Magnetic Resonance Center of Hunan provincial People's Hospital, China. The structure T1-weighted image was acquired without gap, and the number of slices = 176, repetition time = 2,000 ms, echo time = 2.26 ms, field of view = 256 × 256 mm^2^, flip angle = 8°, matrix size =256 × 256, slice thickness = 1 mm.

### Data Analysis

All subjects' structural T1-weighted image data was processed using the statistical parametric mapping software (SPM12, https://www.fil.ion.ucl.ac.uk/spm-statistical-parametric-mapping/) on the Matrix Laboratory MATLAB R2010a platform (Mathworks, Sherborn, MA, USA) according to the following steps: (1) Segmented: the structural T1-weighted image were segmented into gray matter (GM), white matter, and cerebral-spinal fluid based on the “new-segment” tool implemented in SPM8, and both the native space and DARTEL (Diffeomorphic Anatomical Registration Through Exponentiated Lie) imported versions of the tissues were generated. (2) Custom template creation: DARTEL was applied to conduct spatial normalization (11). This procedure achieves more accurate inter-subject registration of images by registering individual structural images to an asymmetric customized T1-weighted template based on different samples, rather than a standard T1-weighted template. (3) Smoothing: smoothed (8 mm full-width at half-maximum, FWHM), modulated, and spatially normalized gray matter images were generated using the Montreal Neurological Institute (MNI) template.

### Statistical Analysis

The bilateral insular cortex was selected as a mask from the automated anatomical labeling (AAL) template using WFU_Pickatlas software (12, 13). A two-sample *t*-test of the insular cortex volumes was conducted in SPM12 using a full factorial model with age, gender, educational level, the number of cigarettes smoked per day and whole brain volume as covariates. All statistical maps were thresholded at *p* < 0.05 with family-wise error (FWE), and a minimum cluster size of 50 voxels was significant. Cerebral regions displaying significant differences in the two-sample *t*-test between the two groups were identified as ROIs (ROI: region-of-interest). The mean volumes of the ROIs were measured for the subjects with craving using the MATLAB program. The Pearson correlation analysis test was performed to investigate the correlation between age, years of education, craving score, and the mean volume in each ROI using SPSS 20.0.

#### ROC Analysis

The receiver operating characteristic (ROC) curve is a graphical plot that summarizes the sensitivity and specificity of a marker for distinguishing subject groups. We applied ROC analysis to evaluate the sensitivity and specificity of the mean gray matter volume changes in the ROIs in order to assess their performance in distinguishing the two groups. As a lot of subjects lost to follow at the second MRI scan, the total of 30 follow-up subjects separated from others. Therefore, subjects were divided into two groups: the model (training) group (*n* = 112, lost to follow) and the validation group (*n* = 30, followed-up). Using area under the ROC curve (AUC) in the assessment of classification model performances is sometimes biased for small sample sizes, and different cross-validation approaches are often used for model validation (14–16). To mimic more datasets of model groups, 112 patients were further randomly divided into four groups of 28 patients and repeated for 30 random partitions (total 120 groups). The ROC analysis was performed for the IC GMV obtained in the model group to evaluate diagnostic performance for detecting craving state. The 2 × 2 contingency tables were constructed for the validation group based on IC GMV smaller vs. larger than the optimal cut-off values and a corresponding number of MADs without vs. with craving, and non-relapse vs. relapse to calculate sensitivity and specificity.

## Results

### Demographic and Clinical Characteristics

Demographic and drug use characteristics of groups of MA users with and without MA craving are shown in Table 1. It shows that there were no significant differences in age, gender, education levels, age of first use MA and smoking history between these two groups. All methamphetamine dependent (MAD) subjects were cigarette smokers. The MA craving group had an average of 4.5 ± 1.84 (mean ± SD) scores in MA craving by VASc from 1 to 10 (low to high). Fifty percentage of them reported medium to high levels of MA craving (scored ≥ 5). Among 30 subjects who returned for a 4-month follow-up, 10 of them had relapsed. The average MA abstinence day for relapsers was 34.0 ± 3.41 (mean ± SD) days.

**Table 1:** Demographic and drug use characteristics between groups of subjects with and without MA craving.

**Characteristic (Mean ± SD)**	**Subjects with MA craving (*n* = 78)**	**Subjects without MA craving(*n* = 64)**	***P*-value**
**Demographics**			
Age (years)	29.0 ± 5.17	29.4 ± 5.91	0.64^*^
Education (years)	11.8 ± 2.63	11.6 ± 2.58	0.70^*^
Male/Female, No.	68/10	56/8	0.95^#^
**MA Use and smoking characteristic**			
Craving scores	2.4 ± 2.61	0	
Age of first MA use (years)	23.7 ± 7.10	24.4 ± 6.21	0.39^*^
Smoking duration (years)	11.5 ± 4.78	11.8 ± 6.45	0.71^*^
Cigarettes smoked per day	20.5 ± 10.04	19.0 ± 10.39	0.38^*^

# *Indicates p-values for Chi-squared test*.

* *Indicates p-values for two-sample t-tests*.

*SD, standard deviation*.

### Decreased Gray Matter Volumes in the Bilateral Insular Cortex in MAD Subjects Without MA Craving

**Figure 1:**
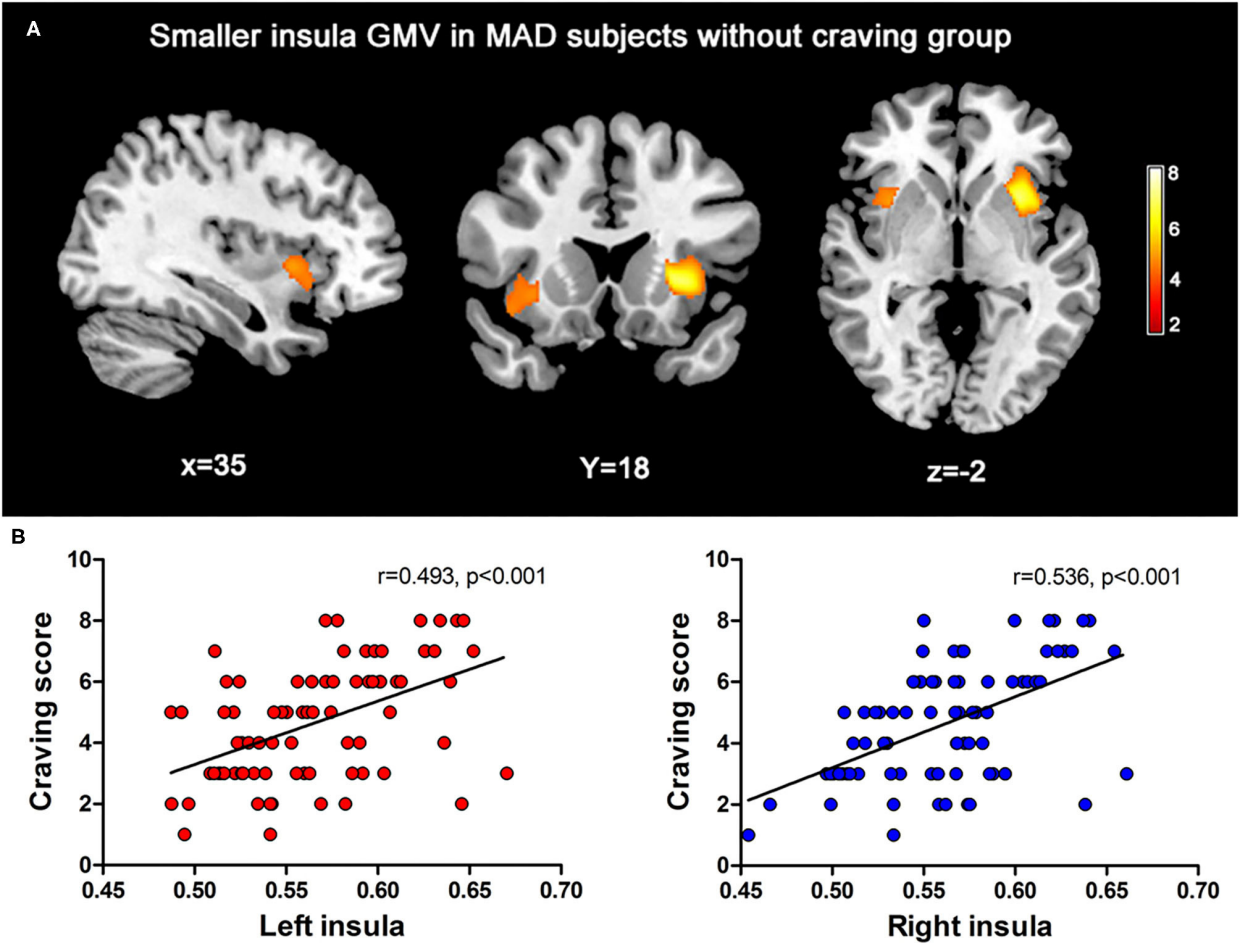
**(A)** The maps for the MADs without craving had extensive significantly decreased in IC GMV (FWE correction, *p* < 0.05) than the MADs with craving. **(B)** The scatter plot of the correlation obtained between regions of interesting of IC GMV with the craving scores reported by the MADs at the first MRI scan.

By voxel-based morphometry (VBM) analysis, a two-sample *t*-test revealed significant differences in the GMV of the IC between the two groups. The group of MAD subjects without craving had decreased GMVs in the bilateral IC compared with the group of subjects with MA craving, which are shown in Figure 1A. Details of smaller gray matter volume (GMV) in subjects without MA craving compared to subjects with MA craving are also shown in Table 2.

**Table 2:** Smaller GMV in MAD subjects without MA craving compared to subjects with MA craving.

**Brain region**	**Cluster size (No. Voxel)**	**MNI coordinates (mm)**			***t*-statistic**
		**X**	**Y**	**Z**	
Left insula	276	−36	−21	10	5.61
Right insula	87	39	18	−12	5.22

*GMV, gray matter volume; MAD, methamphetamine dependence; MA, methamphetamine*.

*Minimum cluster size of voxels: 50*.

*Family-wise error correction, p < 0.05*.

### Decreased Insular GMV Among MAD Subjects Without MA Craving

Among the two brain regions selected as ROIs, the MAD subjects without MA craving group (left: 0.55 ± 0.04; right: 0.54 ± 0.03) showed significantly lower (*p* < 0.05) mean of gray matter volumes in the insular cortex (IC) relative to the MADs with craving group (left: 0.59 ± 0.04; R: 0.58 ± 0.03). As shown in Figure 1B, a positive correlation was found between the volumes of the IC and the craving score in the MADs with craving group (left: *r* = 0.493, *p* < 0.001; right: *r* = 536, *p* < 0.001).

### Detecting Craving State and Predicting Relapse in MAD Subjects

**Figure 2:**
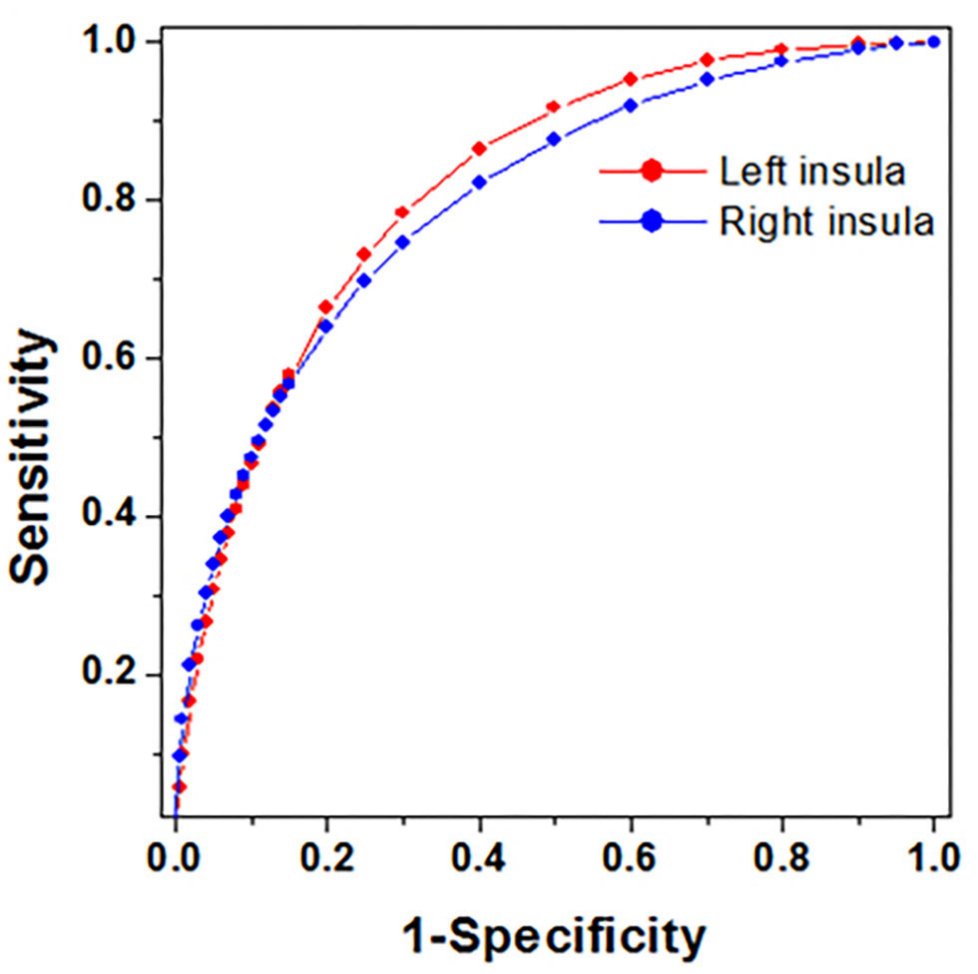
The plots of ROC curves obtained from whole model group MADs. All the sensitivity and specificity were calculated based on Youden index cut-off point.

The ROC analysis for 120 random 28-patients model groups and also for whole model group MADs (Figure 2) showed that there was a good discrimination [area under the curve (AUC)=0.82/0.80 left/right] for IC GMV between the MADs with and without craving. By selecting the Youden index cut-off point from the whole model group, calculated sensitivity/specificity was equal to 78/70% and 70/75% for left and right IC, respectively.

By applying the above optimal cut-off values to 30 follow-up MADs as validations, 2 × 2 contingency tables were constructed for MADs with and without craving (reported at the 1st MRI, Table 3A), and relapse and non-relapse (evaluated at the 2nd MRI, Table 3B). The results from the contingency tables showed a very similar sensitivity (73–80%) and specificity (73–80%) for detecting craving state as a model group. However, there was a low sensitivity (50–55%) and high specificity (80–90%) for predicting relapse susceptibility.

**Table 3A:** The number of follow-up MADs had IC GMV smaller and larger than the optimal cut-off values, and corresponding number of self-reported craving scores at the 1st MRI scan.

**(Left IC, Right IC) 1st MRI**	**Craving state @ 1st MRI**	
	**VASc = 0**	**VASc > 0**
Smaller than cutoff value	(11, 12)	(3, 4)
Larger than cutoff value	(4, 3)	(12, 11)

**Table 3B:** The number of follow-up MADs had IC GMV (measured at 2nd MRI) smaller and larger than the optimal cut-off values, and corresponding number of MADs with relapse and non-relapse.

**(Left IC, Right IC) 2nd MRI**	**Relapse**	
	**Non-relapse**	**Relapse**
Smaller than cutoff value	(11, 10)	(1, 2)
Larger than cutoff value	(9, 10)	(9, 8)

## Discussion

The current study provides the first preliminary evidence that structural MRI could be used to diagnosis the craving state in subjects with methamphetamine dependence (MAD).

We found decreased GMV in the bilateral insular cortex in MAD subjects without MA craving, compared with those who experienced MA craving. Furthermore, we found a positive correlation was found between the GMV of the bilateral IC and the craving score in the MADs with craving group. The gray matter reduction in the bilateral insular in MA dependent patients who experienced no MA craving may indicate the damage to this brain region after long-term use of MA. Among cigarette smoking patients with stroke, the damage to the insula led to an abrupt and profound disruption of dependence on cigarette smoking (7). On the one hand, the decease of drug craving could be considered as evidence of a “cure” for addiction. On the other hand, it may represent the damage of the brain among these chronic drug users. While the high levels of drug craving may represent the less damage to the brain in MA or other drug dependent subjects.

These results stimulate questions about why and how the insula plays an important role in addiction. There has been an increasing number of preclinical and clinical studies exploring the underline specific neural mechanisms about the functions of the insula in addiction. A brain functional connectivity study found that, examination of connectivity with left insula revealed increased connectivity during smoking cue related craving (by smoking videos) compared with food cue related craving (food videos) in the right anterior and posterior insula (17). However, a resting state functional magnetic resonance imaging (rs-fMRI) study assessed changes of resting state connectivity in the insula by treatment of repetitive Transcranial Magnetic Stimulation (rTMS) among treatment-seeking alcohol dependent subjects, and showed no efficacy of rTMS targeting the insula in alcohol addiction (both rTMS and control groups showed a reduction of heavy drinking) (18).

In this study, structural MRI is also used to detect craving state and predict relapse in MAD subjects. The ROC analysis showed that there was a good discrimination (with high sensitivity and specificity) for the GMV of the IC between the MADs with and without craving. Also, there was a high specificity but low sensitivity for predicting relapse susceptibility. The insula has been linked to conscious interoception, emotional experience, and decision- making. A critical underlying theme of this conceptual framework is that a primary goal for the addicted drug users is to obtain the effects of the used drug, and the insula may contribute to how drug users' feeling, memory, and decision about taking drugs (19). A line of research demonstrated the role of the insula in nicotine (20), alcohol (21) and drug (22) addictive behavior. Furthermore, the insula also works as a control system in addiction, involving in weighing the pursuit of certain rewards against uncertain negative consequences, e.g., deciding to take drug or not (23). The current results raise an assumption that if the structure of the insula has been damaged, MA dependent patients' ability of to feel, remembering, or decide to take MA may be disabled. Correspondingly, their craving for MA has been decreasing. However, in addition to neural predictors, there are also clinical and biological factors that predict relapse risk for drug relapse (24). Thus, this study showed low sensitivity for predicting relapse susceptibility.

## Limitations

This study had several limitations. First, only about 20% (30/142) of subjects were included in the 4-month follow-up. In other words, the majority of MA users in this study were lost to follow. Second, this study only included subjects with of MA dependence history. We didn't compare insular GMV between MAD subjects and healthy control subjects who had no drug use history. Third, we only assessed self-reported MA craving by the Visual Analog Scale for craving, and assessed craving as a state rather than a trait. A comprehensive assessment of psychological (as a state) and physical craving (as a trait) should be more accurate. Furthermore, subjects in this study were from drug rehabilitation centers after detoxification treatment. Thus, they may experience less craving for MA than those MA users from the general population. Fourth, the two groups of subjects were matched in age, gender, education levels, age of first use MA, years of cigarette smoking and cigarettes smoked per day (a pack per day on average). However, this study didn't assess nicotine dependence and the impact of nicotine dependence on insular GMV. Fifth, this study only recruited <15% female subjects. So, we didn't assess gender differences in the alternation of insular GMV among MA users. In the future, we will conduct a larger sample follow-up study to investigate how common, specific insular and other brain regions' structural features in MAD patients with and without MA craving are related to clinical implications.

## Conclusion

Our study provides the first preliminary evidence that structural MRI could be used to diagnosis the craving state in MAD subjects based on optimal cut-off values, which could be served as MRI bio-markers and an objective measure of craving state. The smaller IC GMV could alter the insula network, which is critical for the loss of addiction. Although the structural MRI had low sensitivity to diagnose non-relapse, it had high specificity for predicting of relapse. These results from the study may provide valuable insight into clinical diagnosis and treatment for MA and other drug users.

## Data Availability Statement

The raw data supporting the conclusions of this article will be made available by the authors, without undue reservation.

## Ethics Statement

The study protocol was approved by the by the Ethics committee of the Second Xiangya Hospital, Central South University, Hunan, China (No. S095, 2013) and carried out in accordance with the Declaration of Helsinki. The patients/participants provided their written informed consent to participate in this study.

## Author Contributions

CQ and YL conceived the study, did the literature review, and drafted the report. CQ, XF, and SF did statistical analyses. CQ, QW, AX, and ZF collected the data. YL took the lead in writing the manuscript. JT, WH, JL, and TL interpreted the data and commented on the manuscript. All authors contributed to the article and approved the submitted version.

## Conflict of Interest

The authors declare that the research was conducted in the absence of any commercial or financial relationships that could be construed as a potential conflict of interest.
